# Health outcomes of older adults in long-term care homes following hospitalization for hip fracture surgery

**DOI:** 10.1093/geroni/igaf122.3028

**Published:** 2025-12-31

**Authors:** Tiffany Yu, Maya Murmann, Nicholas Yee, Benoît Robert, David Kirkwood, Richard Perez, Amy Hsu

**Affiliations:** University of Ottawa, Ottawa, Ontario, Canada; Bruyère Health Research Institute, Ottawa, Ontario, Canada; University of Toronto, Toronto, Ontario, Canada; University of Ottawa, Ottawa, Ontario, Canada; ICES McMaster, Hamilton, Ontario, Canada; ICES McMaster, Hamilton, Ontario, Canada; Bruyère Health Research Institute, Ottawa, Ontario, Canada

## Abstract

Hip fractures are among the most common and debilitating conditions in the older adult population, particularly for those living in long-term care (LTC) homes. These fractures can reduce quality of life and increase mortality risk. However, surgical management carries potential complications. As a result, older adults and their families often confront the difficult decision of whether to undergo surgery. In this study, we compare outcomes of hospitalized older adults (65+ years old) in Ontario LTC homes that did or did not undergo hip fracture surgery. Data was obtained through the Institute for Clinical Evaluative Sciences (ICES) from 2015 to 2019. Older adults were categorized based on presence of a recorded surgical code. Following this, baseline characteristics and post-discharge outcomes, including mortality, were retrieved from ICES. Outcomes were further stratified by various frailty indices. Among 5,279 older adults meeting inclusion criteria (mean age: 87.01 years, 74.2% female), 4,547 (86.1%) underwent surgery. At baseline, non-surgically managed older adults had higher rates of osteoporosis, hypertension, renal failure, as well as greater functional impairment. Six months post-discharge, older adults who were surgically managed reported fewer deaths in the hospital (4.7% vs 12.8%, p < 0.0001), were less likely to experience frequent pain (6.6 vs 7.3%, p < 0.0001), and saw improved functional independence (13.8% vs 11.9%, p < 0.0001). However, they were also more likely to be physically restrained (11.5% vs 6.3%, p < 0.0001). These findings highlight key factors that influence the decision to undergo hip fracture surgery, which offers invaluable insights for clinical decision-making and care planning in LTC.

